# Understanding Diffusion in a Single-Metal Organic Framework Crystal Used for Sensing Applications

**DOI:** 10.3390/s24123842

**Published:** 2024-06-14

**Authors:** Surya Cheemalapati, Karthik Konnaiyan, Yao Chen, Shengqian Ma, Anna Pyayt

**Affiliations:** 1 Department of Chemical, Biological and Materials Engineering, University of South Florida, Tampa, FL 33620, USA; sekhar.cheemalapati@gmail.com (S.C.); karthikrajk@usf.edu (K.K.); 2 Department of Chemistry, University of South Florida, Tampa, FL 33620, USA; yaochen@usf.edu (Y.C.); sqma@usf.edu (S.M.)

**Keywords:** metal–organic framework, sensing, vitamin B_12_

## Abstract

Metal–organic frameworks (MOFs) stand out as remarkable materials renowned for their exceptionally high surface area and large number of pores, making them invaluable for diverse sensing applications including gas, biomedical, chemical, and optical sensing. Traditional methods of molecule infusion and release often involve a large number of crystals with varying shapes and sizes, leading to averaged outcomes across a heterogeneous crystal population. In this study, we present continuous monitoring of the infusion and release dynamics of model drug molecules, specifically vitamin B_12_, within individual Tb-mesoMOF crystals. Our findings underscore the critical influence of crystal size and shape on the infusion and diffusion processes and corresponding color change, underscoring the necessity to account for these factors in the design of large-scale systems. Leveraging optical microscopy, we employed a histogram-based algorithm for image processing, enabling automated tracking of diffusion phenomena. This investigation offers crucial insights into the dynamics of these processes, laying the groundwork for optimizing parameters in future sensing systems.

## 1. Introduction

Metal–organic framework (MOF) materials have many unique properties that make them very attractive for sensing applications: they have an extremely high surface area, tunable pore sizes and characteristics, and an easy-to-functionalize surface [1,2,3,4]. MOFs are used for a variety of material characterization techniques. In combination with non-destructive dielectric and impedance spectroscopy (IS) techniques used for the detection of the self-diffusion of polar species within porous materials, MOFs are used for the analysis of extrinsic interfacial polarization effects that result in an apparent “colossal” dielectric constant at room temperature [5]. The diffusion process can be especially complicated when there are multiple pore sizes present in the same MOF [6]. Monolithic MOF thin films, i.e., surface-mounted metal–organic framework (SURMOF) films are widely used as model systems for studying molecular interactions in porous solids [7]. The individual crystals can be synthesized reproducibly as large as millimeter size, which aids in the accurate measurement of micropore diffusion coefficients [8].

Traditionally, the analysis of molecular uptake and release in MOFs has relied on batch averaging techniques, such as gravimetric tests [9,10,11,12], elemental analysis [13], inductively coupled plasma mass spectrometry (ICP-MS) [14], ultraviolet–visible (UV–VIS) spectroscopy [9,11,13], absorbance [12,13,15,16], high-performance liquid chromatography (HPLC) [9,11], fluorescence measurements [9,11,14,16], nuclear magnetic resonance [17], physisorption analyzers, and quasi-elastic neutron scattering [18]. However, it is recognized that single-crystal guest-molecule concentration profiles differ from average bulk uptake/release data [19] due to the presence of defects such as cracks [20], pore clogging [21], or external effects [22]. Additionally, within a single crystal, there exist preferred directions in the crystal lattice, leading to faster diffusion of guest molecules through certain faces. Hence, single-crystal studies are imperative for a deeper understanding of processes occurring at the individual crystal level. Previous single-crystal studies have relied on interference microscopy [23,24] and Fourier-transform infrared spectroscopy (FTIR) [24], necessitating complex experimental setups and analyses.

Previous studies have demonstrated that the maximum load of vitamin B_12_ per mg Tb-mesoMOF is 0.33 mg [25]. In a sensing system, this will define the maximum detection limit. Since the color change does not occur uniformly across the crystal during the early infusion time (20–60 min), colorimetric detection would not work for low concentrations. However, absorption measurements averaging across the whole batch would work efficiently. Additionally, early detection (within a 60 min time limit) is desirable for the following reasons: (1) it is better to conduct the measurements quickly to eliminate delays and limit the cost of sensing, and (2) the initial uptake can be approximated by a linear function (as a small section of a parabola), simplifying the measurements and not introducing significant error. The total initial uptake was approximately 11.2% per hour [26]. This can be followed by quantifying vitamin B_12_ using absorption spectroscopy with a detection limit of 10 ng/mL [26].

Here, we introduce a new technique for analyzing molecular transport in a single MOF crystal. We employ optical microscopy [27] to investigate the uptake and release of red-colored molecules of cobalamin (vitamin B_12_), an essential drug molecule administered to prevent neurologic and psychiatric disorders [28]. Tb-mesoMOF was synthesized through the coordination of TATB ligands and Terbium, resulting in a structure with hierarchical mesoporous pores. Tb-mesoMOF is a colorless crystal, making it easy to track and observe the encapsulation of colorful guest molecules in this system. The two types of cages are a 3.9 nm diameter cage and a 4.7 nm diameter cage interconnected through 1.3 and 1.7 nm windows in Tb-mesoMOF [29]. The dimensions of the vitamin B_12_ molecule are 1.41 × 1.83 × 1.14 nm [25]. In this study, vitamin B_12_ was diffused into a Tb-mesoMOF crystal by immersion in a vitamin B_12_–methanol solution. Visible images were captured at the beginning and regular intervals to elucidate the phase-supported diffusion of guest molecules. Subsequently, a fully infused MOF crystal was immersed in pure methanol to initiate molecule release, with continuous monitoring facilitated by optical microscopy. Images extracted from the video footage were processed to obtain the transient concentration profile of the drug within a single MOF crystal.

## 2. Materials and Methods

First, freshly synthesized Tb-mesoMOF crystals were characterized using optical and scanning electron microscopy (HITACHI SU-70 SEM produced by Hitachi Ltd., Tokyo, Japan) with an accelerating voltage of 15 kV). Optical images of the crystals were taken with an optical microscope Nikon FN1 (produced by Nikon, Tokyo, Japan) operated under bright field conditions, with a CFI Plan Fluor 10× Objective Lens NA 0.3 WD 16MM, Eco-Glass (Produced by Nikon, Tokyo, Japan). Optical images of the crystals were taken by an Olympus MIC-D camera (produced by Olympus, Tokyo, Japan) with Acquisition and an analysis software package, NIS Elements 4.5 (developed by Nikon, Tokyo, Japan), with SOLA SM II LE light providing broad white output with no filters. Crystalline samples of the Tb-mesoMOF were prepared according to the procedures [29]: Tb(NO_3_)_3_⋅5H_2_O (0.030 g, 6.90 × 10^−5^ mol) and 4,4′,4″-s-trizaine-2,4,6-triyltribenzoic acid, H3TATB (0.010 g, 2.27 × 10^−5^ mol) were dissolved in DMA/MeOH/H2O (2.0/0.4/0.1 mL) in a 20 mL vial at ambient temperature [25,30]. The reaction mixture in a capped vial was heated in an oven at 105 ºC for 2 days [25,30]. It was previously published that the freshly synthesized Tb-mesoMOF crystals were characterized by powder X-ray diffraction (PXRD) using a Bruker (Billerica, MA, USA) D8 Advancedtheta-2theta diffractometer with copper radiation (Cu Ka, l = 1.5406 Å) and a secondary monochromator operating at 40 kV and 40 mA [30,31]. Also, PXRD confirmed the integrity of the MOF after encapsulation [25,30,31].

Then, experiments regarding diffusion into (uptake) and diffusion out of (release) red-colored guest molecules (vitamin B_12_ in Tb-mesoMOF) were conducted. To monitor the uptake of vitamin B_12_ into Tb-mesoMOF, multiple crystals were placed in a Petri dish containing a vitamin B_12_ solution (concentration 0.01 g/mL). The solution had an intense red color; thus, the diffusion of molecules into the crystal could be visualized with a color map showing both distribution and intensity. The uptake was carried out in a solution for 12 h at room temperature. Optical microscopy and scanning electron microscopy images were taken to monitor the diffusion process and understand physical parameters that could influence the transport of the molecules in the crystal. Crystal color information was extracted from optical images, while the nano-scale structure, surface properties, and defects were observed using SEM.

After that, the release of vitamin B_12_ from the crystal was initiated and monitored by placing several fully infused crystals in methanol and continuously recording changes of the crystal color using a camera attached to an optical microscope. Since release takes a significantly longer time than uptake, diffusion of the vitamin B_12_ out of the Tb-mesoMOF crystal into methanol took ~7000 min (~116.7 h). An image processing algorithm was developed to extract information about diffusion dynamics from the video using histograms.

**Figure 1:**
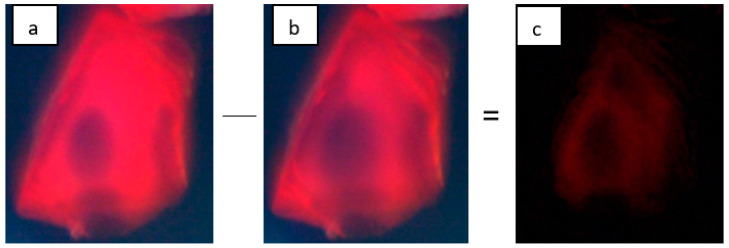
First step in image processing. Image (**b**) at is subtracted from initial image (**a**) to produce the differential image (**c**).

**Figure 2:**
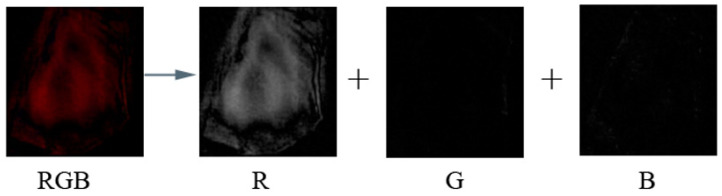
Step 2 in image processing where RGB values of subtracted images are extracted.

The color histogram is a vital parameter in image processing that can be used in various applications such as calculating concentrations of dye inside the nucleus of a cell during specific time intervals [32] and finding out the various hematological conditions by using red blood cell histogram values [33]. In another study, grayscale histograms of MRI images were used to determine the grade of glioma by calculating the apparent diffusion coefficient. A wide spread of the histogram indicates a severe tumor condition [34]. A similar methodology is used to calculate the diffusion rate of the MOF crystals. Mean intensity values of the total pixels in the images are calculated and plotted. Mean intensity value indicates the relative concentration of the vitamin B_12_ that has diffused out of the MOF crystal. Image processing entailed the following steps:

Step 1: Images of B_12_-infused crystals at different time intervals were collected and analyzed. The first image was used as the reference. It was subtracted from all consecutive images. An example is shown in Figure 1 below. The intensity values of the subtracted images are used to visualize the change in diffusion at different time intervals;

Step 2: The differential image (Figure 2) has 3 color channels, red, green, and blue (RGB), as shown in the figure below. Since B_12_ is red, the red image channel was used for further analysis. It was converted to a grayscale image representing 1 as black and 255 as white, encoding the red color’s intensity information. A custom program was created to extract the red-colored plane information from the RGB image and calculate the mean value of the image histogram; then, it was plotted as color difference intensity vs. time.

After that, we developed a procedure for calculation of the diffusion coefficient. In general, a diffusion coefficient can be calculated by using this equation [35]:

$$x^{2}=q_{i}\times D\times t$$

where *x*^2^ is mean-square displacement (*x* is the mean distance from the starting point that a molecule has to diffuse in time *t*); *q_i_* is a constant, which depends on the dimensionality of diffusion analysis *q_i_* = 2, 4, or 6, for 1-, 2-, or 3-dimensional diffusion; *D* is the diffusion coefficient (cm^2^ s^−1^); and *t* is the time (s).

In this paper, for the diffusion coefficient calculation, we use a mathematical apparatus like the one used for fluorescence recovery after photobleaching [FRAP]. A two-dimensional diffusion coefficient (*D*) can be calculated using the equation from [36,37,38]:

$$D=\frac{\omega^{2}}{4t_{D}}$$

where *ω* is the change in diameter of the photobleached spot (for FRAP) or linear change of the spot that had diffused out in our experiments (as discussed at the end of the Section 3), and *t_D_* is the characteristic time [37,38] that it takes the signal to change to 50% of its initial value.

## 3. Results

**Figure 3:**
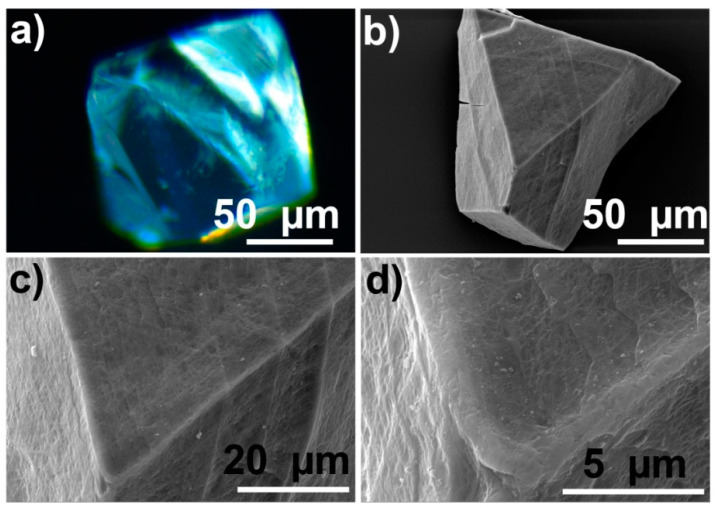
Optical and SEM characterization images of individual crystals of Tb-mesoMOF before infusion with vitamin B_12_: (**a**) Optical microscope image of the MOF; (**b**) SEM image of MOF and (**c**,**d**) are higher magnification parts of the crystal shown in (**b**).

Figure 3a shows an example of a MOF crystal before infusion. This optical microscopy image demonstrates typical a Tb-mesoMOF crystal with linear dimensions ~100 µm. Different crystal faces are well defined, and the color information can be easily extracted. The original crystal is clear before any infusion, and thus vitamin B_12_-related color change is very pronounced. The scanning electron microscope (SEM) image of a similar crystal, Figure 3b, shows more structural details because of the larger depth of field and higher resolution. Figure 3c,d show the same crystal with higher magnification. It can be noticed that the crystal has very well-defined crystal planes with many more details than that in an optical microscopy image. Additionally, even though it looks smooth under the optical microscope, the higher magnification SEM image (Figure 3d) shows that it has nanoscale surface roughness. Below is a comparison of two types of information—color from optical microscopy and structure from SEM are used to study mechanisms of molecular infusion in single Tb-mesoMOF crystals and identify the factors that influence this process.

**Figure 4:**
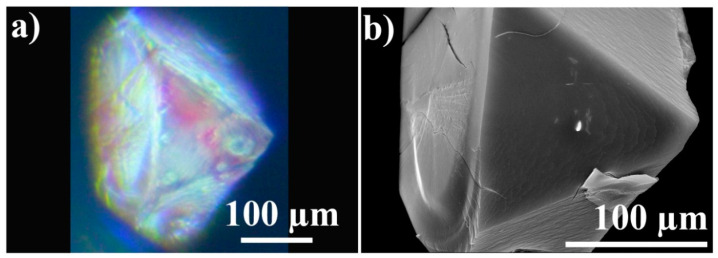
Optical microscopy and SEM characterization of vitamin B_12_ uptake in Tb-mesoMOF: (**a**) Optical microscope image of a crystal infused with vitamin B_12_ for a short period of time; (**b**) corresponding SEM image of the same crystal in the same orientation as in the optical microscopy image.

Next, the analysis of the crystal shape was followed by the microscopic study of diffusion into MOF crystals. It was conducted through a series of experiments. First, early diffusion dynamics were investigated. A Tb-mesoMOF crystal was incubated in the solution of the vitamin B_12_ for nearly 30 min and then was taken out and observed under optical microscope (Figure 4a). Due to the intense red color of the vitamin B_12_ solution, diffusion of the molecules into the crystal could be tracked by monitoring the crystal as it partially changed its color to red.

**Figure 5:**
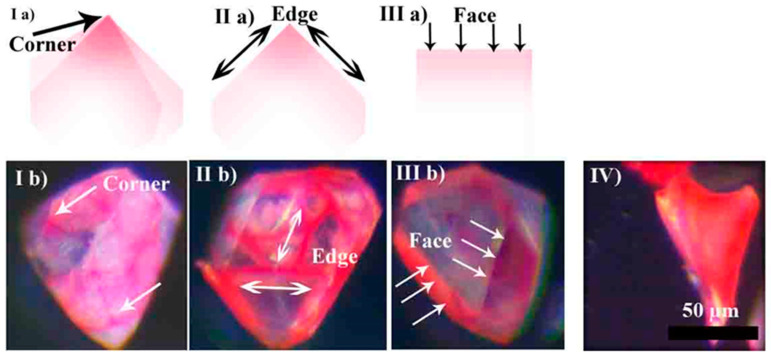
Optical microscopy monitoring of dynamics of molecular vitamin B_12_ uptake by individual Tb-mesoMOF crystals. (**Ia**,**IIa**,**IIIa**) are schematic representations of microscopy images taken at 20 min (**Ib**), 60 min (**IIb**), and 300 min (**IIIb**), respectively, and (**IV**) is an image taken at 780 min, during the final stages of uptake.

The SEM image, Figure 4b, shows the structural details that cannot be seen on the optical image and can be used to explain the irregularities of the color during early molecular uptake. It can be noticed that molecular uptake is happening faster in the areas with 3D defects: broken away pieces, bumps, corners, and scratches look significantly more red than flat smooth areas. It is caused by molecular intake from multiple directions and is similar to the observations demonstrated in Figure 5.

The influence of the structural elements of the crystal on molecular uptake is shown in Figure 5. It demonstrates that the presence of multiple access planes significantly speeds up the uptake. Figure 5(Ia,Ib) show schematics and experimental demonstration of the intense color change in the corners of the crystal. While in the whole crystal body the color change is negligible, the corners can be seen as bright spots after just 20 min in vitamin solution. After 60 min, all edges become bright, since in those areas diffusion is going through two neighboring faces (Figure 5(IIa,IIb)). After 300 min, the crystal is noticeably red, since the even slower one-face-based diffusion is producing enough color change. Figure 5IV demonstrates a crystal that was incubated for 780 min and from the outside looks uniformly red.

**Figure 6:**
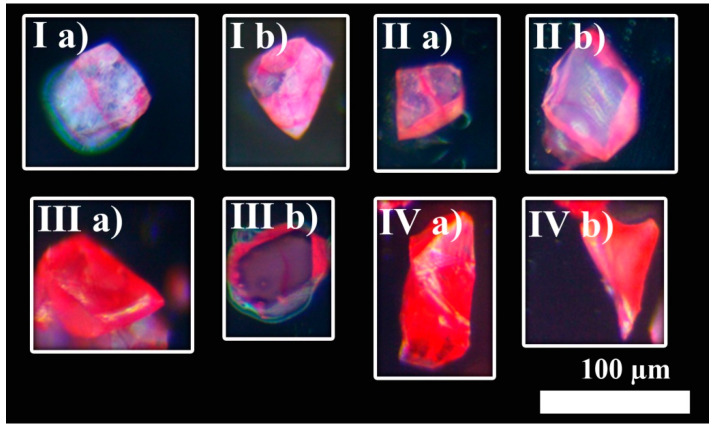
Optical microscopy monitoring of dynamics of molecular vitamin B_12_ uptake by individual Tb-mesoMOF crystals. Image (**Ia**,**Ib**) show uncut samples of MOF infused with vitamin B_12_ for 10 min. (**IIa**) shows uncut, and (**IIb**) shows cut MOF crystals infused with vitamin B12 for 20 min. (**IIIa**) shows uncut, and (**IIIb**) shows cut MOF crystals infused with vitamin B_12_ for 180 min. Here, the MOF looks red from outside, but the cut sample shows there is still vacant MOF. (**IVa**) shows uncut and (**IVb**) cut MOF crystals infused with vitamin B_12_ for 780 min. Here, the MOF is completely infused.

After that, continuous dynamics of vitamin B_12_ uptake were studied using multiple Tb-mesoMOF crystals placed in vitamin B_12_ solution. Crystals were taken out of the solution at regular intervals and observed under an optical microscope (Figure 6). Some of them were studied directly to obtain information about surface distribution of the guest molecules. Others were cut in half to study volume distribution. Figure 6 demonstrates that the intensity of the red coloration of individual crystals increases with time and progresses from pale pink in Figure 6(Ia) to a bright red color in Figure 6IV. However, this color change did not happen uniformly across the whole batch of the single crystals. For example, both crystals shown in Figure 6(Ia,Ib) were infused for 10 min. However, the crystal shown in Ib has higher surface roughness, a larger number of edges, and more surface defects and thus has a brighter pink color than the crystal shown in Ia.

Sections II–IV have two subsections each—a and b. There, (a) demonstrates samples that were not cut, and (b) demonstrates ones that were split in half. The splitting was performed with a diamond scriber while observing them under the microscope. Figure 6(IIa,IIb) show Tb-mesoMOF crystals after 20 min of vitamin B_12_ uptake. The crystal shown in Figure 6(IIa) has some of the faces completely colored in red, while others are mostly colored along the edges. The second sample shown in Figure 6(IIb) is cut in half, and its interior is still intact and colored white, implying that no or little diffusion reached the middle part of the crystal. Additionally, most of the diffusion into the crystal interior happened through the brightly colored faces. The same effect was observed with a longer diffusion time.

In Figure 6III,IV, the images were taken at 180 and 780 min, respectively. After 180 min, the whole surface of the crystal was red (Figure 6(IIIa)); however, cutting the crystal in half and looking at its interior demonstrated that diffusion into the crystal happened at different speeds along different faces forming well-defined layers of vitamin B_12_-infused material. After 780 min, crystals were completely infused and looked red from the outside (Figure 6(IVa)) and inside (Figure 6(IVb)).

**Figure 7:**
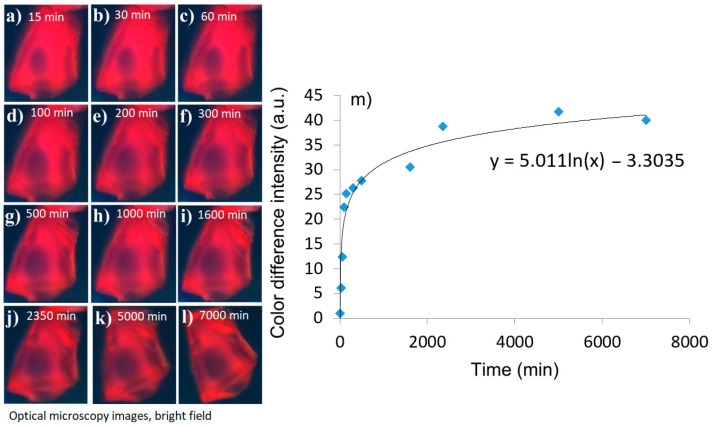
Optical microscopy characterization of vitamin B_12_ release from an individual Tb-mesoMOF crystal. Images of the crystal infused with vitamin B_12_ suspended in methanol, and vitamin B_12_ diffusion out of the crystal at times (**a**) t = 15 min, (**b**) t= 30 min, (**c**) t = 60 min, (**d**) t = 100 min, (**e**) t = 200 min, (**f**) t = 300 min, (**g**) t = 500 min, (**h**) t = 1000 min, (**i**) 1600 min, (**j**) t = 2350 min, (**k**) t = 5000 min, and (**l**) t = 7000 min; (**m**) a plot demonstrating speed of release of the vitamin B_12_ vs. time.

Another important property of a sensing system is if it can be reused. The following experiments demonstrate that there are significant limitations for reusability, and ideally fresh batches of crystals should be used every time. Specifically, the next part of the study evaluated molecular release of the vitamin from crystals. It was observed that all release happened within 3000 min, and it stops after that even though the crystal is still colored. A Tb-mesoMOF crystal infused with vitamin B_12_ was placed in methanol, and its color change was monitored continuously for 7000 min (4.8 days) using a camera attached to an optical microscope. Images were extracted from the video at specific time intervals as shown in Figure 7a–l. Initially, the crystal looked red, and there was a small dark region in the middle indicating the area where molecules did not reach during the infusion process. During the drug release experiment, the dark area increased, while the red-colored area decreased. The information about the level of infusion was extracted with image processing using the algorithm below.

It can be noticed that the red-colored area continuously decreases with time indicating diffusion of vitamin B_12_ out of the crystal. All the changes were monitored relative to the initial state of the crystal shown in Figure 7a. The maps of color change were obtained by subtracting the initial image (a) from subsequent images (b through l). Next, histograms were applied to the color analysis of the subtracted images. Here, we adapt this technology to study diffusion dynamics in MOF crystals by using continuous tracking of histogram change of the images of the crystal. Consecutive images taken at different time intervals were digitally subtracted from the reference image, and the resulting difference was used for further estimation of diffusion dynamics. Specifically, RGB (red, green, and blue) components of the subtracted images were separated, and only the red component was used for further analysis. The red component of the image was converted to a grayscale image with 0 being black and 255 as white. Mean intensity values of the images were calculated and plotted (Figure 7m), and the approximation for logarithmic diffusion was used [39]. This change in mean intensity value was correlated to the dynamics of the vitamin B_12_ diffusion out of the MOF crystal. It can be observed that the initial release was high during first 3000 min, but it then reached a plateau and almost stopped, even though the crystal still had areas infused with the vitamin and colored in red. The estimated diffusion rate was calculated based on the mathematical apparatus described in the previous section; it was found to be ~1.49 × 10^−15^ m^2^/s. The order of magnitude of the diffusion coefficient corresponds well with previous findings [19,40,41,42,43,44,45].

## 4. Discussion

The study presented in this paper analyzes dynamics of molecular transport within metal–organic framework (MOF) crystals, focusing particularly on the uptake and release of vitamin B_12_ molecules in individual Tb-mesoMOF crystals. Through a combination of optical microscopy and scanning electron microscopy (SEM), we investigated the infusion and diffusion processes, taking into consideration the role of crystal size, shape, and surface defects in influencing these phenomena.

Our findings support the significance of single-crystal studies in understanding the nuanced behavior of guest molecules within MOF structures. Traditional batch averaging techniques often obscure the details inherent in individual crystal behavior, such as variations in diffusion rates across different crystal faces and the impact of surface defects on molecular transport. By employing optical microscopy coupled with SEM, we were able to analyze this complex dynamic. The experimental results demonstrated differences observed in diffusion rates between various crystal faces. Moreover, surface defects, edges, and corners greatly influence the diffusion process and contribute to heterogeneity in molecular distribution within the crystals. In addition to that, over time (780 min in our experiments), the crystals become fully infused with B_12_. As a result, no further infusion becomes possible since the crystal has reached its full capacity. This observation of the early-infusion non-uniform color change and the late-infusion saturation greatly affects MOF-based sensing at low and high concentrations and limits accuracy and the detection limit. In addition to that, the average size of the MOF would affect the highest detectable level, since the molecules diffuse from the surface to the center, and after the center is reached, the crystal would not be able to accept any more molecules.

Another important goal of this research was to investigate reusability of the same crystal batch. Toward that goal, we analyzed the release kinetics of vitamin B_12_ from fully infused Tb-mesoMOF crystals. We observed a substantial release of the drug molecule within the initial 3000 min, followed by a plateau phase, even though the crystal was still visibly red. This observation suggests that even though the crystals can be potentially reused, the color-based detection must be recalibrated after each use, and the lowest detection limit might be affected after the first use.

The proposed methodology offers a robust framework for optimizing MOF-based sensing platforms, providing insights into the design parameters governing molecular uptake and release. Additionally, the estimated diffusion rate obtained in our study aligns well with previous findings, confirming the accuracy of our approach.

In conclusion, this research contributes to the growing body of knowledge focused on MOF materials and their applications in sensing technologies. By understanding the molecular transport at the single-crystal level, we pave the way for the development of tailored MOF-based sensors with enhanced accuracy, lower detection limits, and increased range. We emphasize that MOF-based sensing systems require optimization of not only the specific MOF compositions but also their size and morphology.

## 5. Conclusions

In this paper, we demonstrated single-crystal studies of uptake and release of guest molecules in MOF using optical microscopy and SEM. Differences in diffusion through different crystal faces were observed. Additional differences in diffusion are caused by surface defects and variability of shape from crystal to crystal. Finally, release of vitamin B_12_ from a single Tb-mesoMOF-100 crystal was investigated, and it was shown that substantial release lasts for ~3000 min, while complete infusion is four times faster. This approach can be used for optimization of MOF-based sensing and provides insight on how to create crystals with desired uptake and release profiles. One of the main recommendations for the use of MOFs in sensing systems is to optimize not only specific types of MOFs but also their size since it is going to significantly influence system characteristics.

## Data Availability

Data is contained within the article.
